# Immunostimulatory and anti-inflammatory impact of *Fragaria ananassa* methanol extract in a rat model of cadmium chloride-induced pulmonary toxicity

**DOI:** 10.3389/fimmu.2023.1297315

**Published:** 2023-11-29

**Authors:** Amany E. Nofal, Hind S. AboShabaan, Reda M. Fayyad, Rafik E. Ereba, Nassar A. Omar, Sherin M. Elsharkawy, Aya I. Elberri

**Affiliations:** ^1^ Histology and Histochemistry Division, Zoology Department, Faculty of Science, Menoufia University, Shebin El-Kom, Egypt; ^2^ Clinical Pathology Department, National Liver Institute, Menoufia University, Shebin El-Kom, Egypt; ^3^ Department of Pharmacology, Faculty of Medicine, Al-Azhar University, Cairo, Egypt; ^4^ Anatomy, Faculty of Medicine, Sohag University, Sohag, Egypt; ^5^ Physiology Department, Medicine College, Benha University, Benha, Egypt; ^6^ Genetic Engineering and Molecular Biology Division, Zoology Department, Faculty of Science, Menoufia University, Shebin El-Kom, Egypt

**Keywords:** Ashcroft scoring, chromatographic analysis, immunomodulatory, NF-κB, Nrf2, oxidative stress

## Abstract

Cadmium is an extremely dangerous heavy metal that can lead to disastrous consequences in all organisms. Several natural remedies reduce the toxicities of experimentally generated metals in animals. Strawberry *Fragaria ananassa* contains several bioactive compounds that may mitigate heavy-metal toxicity. The study aim was to evaluate the ability of a strawberry fruit methanol extract (SE) to reduce Cd toxicity and to identify and quantify the active constituents of SE. Forty Wistar rats were classified into four groups: the control group– 1 ml saline IP; SE group– 100 mg of SE/kg rats orally; cadmium (Cd) group–2 mg CdCl_2_/kg body weight/IP daily; and treated group– SE given 1 hour before Cd administration. Administration of Cd induced several histopathological and immunohistochemical alterations in lung sections. Biochemical analysis of lung homogenates and mRNA levels of antioxidants and inflammatory cytokines indicated significant changes to the risk profile. SE administration significantly decreased the oxidative stress, inflammation, tissue damage, the mean area percentage of collagen fibers, and positive immuno-expressions of TNF-α and NF-κB induced by CdCl_2_. Moreover, the MDA, *TNF-α*, *GM-CSF*, and *IL-1β* levels in Cd-exposed rat lung tissue were significantly lower in the SE-treated group than in the Cd-group. SE significantly augmented lung GSH, SOD, *HO‐1*, *GPx-2*, and *Nrf2* levels in Cd-exposed rats. SE mitigated Cd-caused oxidative stress and lung inflammation. Therefore, regularly consuming a strawberry-rich diet could benefit general health and help prevent and treat diseases.

## Graphical Abstract

Schematic overview how these results may explain the ameliorative effect of SE on the lung injury.

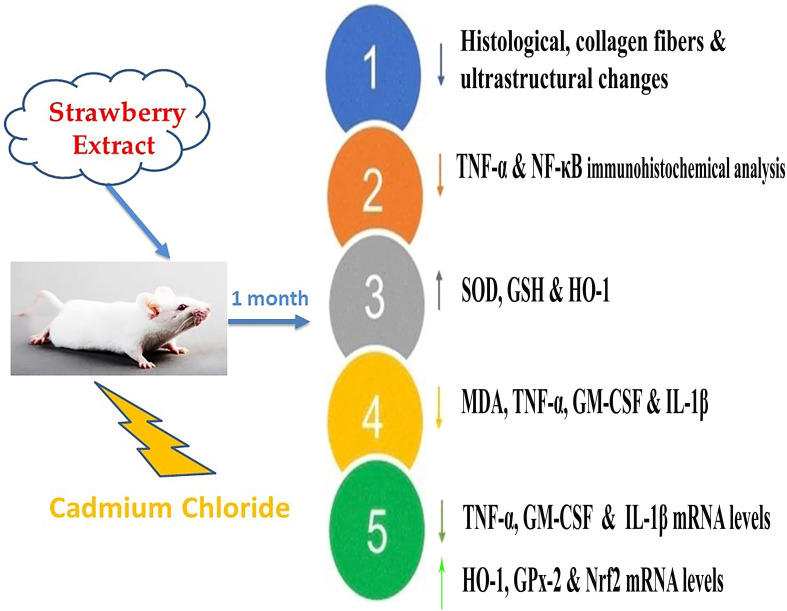

## 1 Introduction

Cadmium (Cd) is a very toxic heavy metal that frequently contaminates the surrounding environment in agricultural and industrial operations. Cd enters the soil, infiltrates the water, and is then easily absorbed by plants and stored in crops (1, 2). Cd can accumulate in the body and causes many disorders in most body organs, including the lungs, liver, kidneys, and cardiovascular system (3, 4). Cadmium poisoning has been recorded in several countries throughout the world. It is one of the worldwide health issues that affects various organs and, in some circumstances, kills people every year. Long-term cadmium exposure through air, water, soil, and food causes cancer as well as organ system damage in the skeletal, urinary, reproductive, cardiovascular, central and peripheral neurological systems, and respiratory systems. Cadmium levels in blood, urine, hair, nails, and saliva may all be tested (5, 6). A study found that Cd enhanced the generation of reactive oxygen species (ROS), which was time- and concentration-dependent, leading to increased lipid peroxidation, membrane protein damage, and DNA loss (7). Cadmium influences cell proliferation and differentiation by producing reactive oxygen species (ROS), which causes oxidative stress and the activation of apoptosis. Furthermore, cadmium causes chromosomal aberrations, DNA strand breaks, mutations, and chromosomal deletions by depleting reduced glutathione (GSH), which increases the production of ROS such as superoxide ion, hydrogen peroxide, and hydroxyl radicals. Furthermore, cadmium inhibits the activity of antioxidant enzymes such as superoxide dismutase and modulates the cellular level of Ca^2+^ as well as the activities of caspases and nitrogen-activated protein kinases in cells, causing apoptosis indirectly (8, 9).

The primary effects of Cd toxicity are oxidative stress and inflammation (10). In exposed cells, apoptosis and necrosis are the ultimate results of Cd poisoning if the healing process was not quickly balanced (11). In populations with persistent exposure to low doses of Cd, ad-verse health effects, such as hypertension, neurotoxicity, type 2 diabetes, and cancer are seen (2, 12).

Generally, it is accepted that a diet supplemented with vegetables and fruits is useful for human health because of its high content of antioxidants and phytochemicals (13, 14). Consequently, increased consumption of strawberries may protect against several chronic illnesses (15, 16). Strawberry phenolic have been documented to exert anticarcinogenic, anti-inflammatory, antiproliferative, and antiatherosclerosis properties (17). Strawberry contains effective anti-inflammatory and antioxidant phytochemicals, including caffeic acid, anthocyanin, ellagic acid, and flavonoids, such as catechin, tannins, kaempferol, quercetin, and derivatives of gallic acid. Additionally, it is rich in vitamins C and E as well as carotenoids (18). Strawberry extract (SE) mitigated the oxidative damage caused by a high-fat diet (17, 19, 20).

The current study aim was to investigate the nutraceutical potential of SE and its ameliorative effect on Cd-induced lung damage as well as to identify and quantify the active constituents of SE.

## 2 Materials and methods

### 2.1 Chemicals

All analytical-grade chemicals, standard phenolic acids, flavonoids, and cadmium chloride (CdCl2) were purchased from Sigma-Aldrich (St. Louis, MO, USA).

### 2.2 Strawberry extract preparation

The fruit of fresh strawberry, *Fragaria ananassa* Duch. (Rosaceae), was obtained from the local market “El-Gelany” of Menoufia in November 2020. The plant identification has kindly been confirmed in the Botany Dep., Menoufia Unv. and a voucher specimen (PG-A-F-W-10) was preserved at Zoology Dep., Menoufia Unv. A commercial blender squeezed the fresh strawberry fruits (1 kg) after washing and removing their leaves, filtered the juice, and then lyophilized it to obtain powdered strawberry. The lyophilized strawberry powder (100 g) was extracted with 70% methanol in water three times. Next, the aqueous extract was evaporated at low pressure. The residue yield was 33.56% and kept at −20°C.

### 2.3 High-performance liquid chromatography analysis

Analysis of the SE was carried out using the HPLC technique in the National Research Centre (NRC), Egypt according to Kunti´c et al. (21). The SE was analyzed using an Agilent 1260 series instrument and Eclipse C18 column (4.6 mm × 250 mm i.d., 5 µm). Separation was carried out at a flow rate of 0.9 mL/min. The mobile phase consisted of reservoir A and B (water and 0.5% trifluoroacetic acid in acetonitrile, respectively) at a concentration of 0.1%. The mobile phase program was sequentially adjusted with a linear gradient as follows: 0 min, 82% A; 0–5 min, 80% A; 5–8 min, 60% A; 8–12 min, 60% A; 12–15 min, 82% A; 15–16 min, 82% A, and 16–20, 82% A. Detection at 280 nm was performed by using a multi-wavelength UV detector. The injection volume for each sample solution was 5 μL. The column temperature was maintained at 40°C.

### 2.4 Experimental design

The current research was approved by the Ethics Committee of the Menoufia University, Egypt (IACUC, Approval No. MUFS/F/HI/6/20).

Forty adult male Wistar rats (Approximately 8 weeks old, 180–195 g) were procured from Helwan University, Egypt, the Animal Care Centre. The rats were housed at 25°C ± 2°C under a light/dark cycle with full access to water and food. The animals were acclimated for 1 week before the experiment started. Then, the animals were divided randomly into four equal groups over the 30-day study period: the normal control group (Con) intraperitoneally (IP) received 1 mL of isotonic physiological solution, the SE group gavage received 100 mg of SE/kg rat body weight (BW) in saline (18); the Cd group received IP-administered CdCl_2_ at a dose of 2 mg/kg BW/daily in saline (10); and the SE+Cd treated group received SE 1 hour before CdCl_2_ administration as above. At the end of the study, the rats were euthanized by Thiopental sodium (N01AF03, ADVANZ Pharma, www.advanzpharma.com) for dissection, and the right lungs were harvested for ultrastructural, histopathological, and immunohistochemical studies. The left lungs were homogenized for the different inflammatory cytokines and antioxidant measurement techniques.

### 2.5 Methods and assays

#### 2.5.1 Histology, ultrastructural, and immunohistochemistry techniques

The right lungs of the various groups were separated, fixed in 10% neutral formalin(252549 Formaldehyde solution 37-40%, Sigma-Aldrich from Schnelldorf, Germany), paraffin-embedded, and sectioned to a thickness of 5 µm. The sections were processed and viewed after staining with hematoxylin and eosin (H&E; H3136 Ehrlich’s Hematoxylin, E4009 Eosin Y, Sigma-Aldrich from Schnelldorf, Germany) for histopathological analysis (22), and with Masson’s trichrome (HT10516 Trichrome Stain AB Solution, Sigma-Aldrich from Schnelldorf, Germany) revealed collagen deposition and pulmonary fibrosis (23). Small lung pieces (1 mm^3^) were processed for transmission electron microscopic study (TEM); they were fixed with formalin-glutaraldehyde fixative (4F1G) in phosphate-buffered saline and then post-fixed in 2% osmium tetroxide in the same buffer for 2 h at 4°C (24). Semithin sections (0.5–1 μm) were cut, stained with toluidine blue (89640 Toluidine Blue, Sigma-Aldrich from Schnelldorf, Germany), and examined under a light microscope. As previously described an LKB ultratome was used to cut ultrathin sections from the selected areas in the semithin sections, mounted on copper grids, and stained with uranyl acetate and lead citrate. They were investigated on a JEOL 100 CX-TEM (J. E. M. 100 CXII) at the Electron Microscope Unit, Alexandria University, Egypt. Lung paraffin sections (4 µm) on positively-charged slides were blocked by avidin–biotin–peroxidase to look for inflammatory cells with immunohistochemical (IHC) reactions to tumor necrosis‐alpha (*TNF‐α;* SAB5700698-100UL Anti-TNF-α polyclonal antibody produced in rabbit, 1:50-1:100, Sigma-Aldrich, USA) (25), and nuclear factor kappa-light-chain-enhancer of activated B cells *(NF-κB;* AV32718-100UL, Anti-NFKB2 polyclonal antibody produced in rabbit, Sigma-Aldrich, USA) (26). Positive reactions appeared as brown reactions, and the cell nuclei were counterstained with hematoxylin (MHS16 Mayer′s Hematoxylin Solution, Sigma-Aldrich from Schnelldorf, Germany).

#### 2.5.2 Images and histological analysis

The lung sections were examined under a microscope and photographed with an Olympus digital camera attached to a light microscope (Olympus BX 41, Japan). Pulmonary histopathological scoring was assessed by using a semi-quantitative technique ranging from 1 to 5, as 5 is the most serious histopathology, the light microscopic analysis of the histological slices was performed as a descriptive analysis of the characteristics seen in the slices (27). Using a semi-quantitative grading method, the digitized photographs were examined (Image J software) for the number of alveolar macrophages and inflammatory cells as well as the percentage of the positive area of histological collagen and then subjected to immunohistochemical analysis. All measurements were taken in 10 randomly selected fields at ×400 magnification from eight rats in each experimental group. In H&E- and Masson’s trichrome-stained lung sections, the interalveolar septa thickness and area percentage of the positive collagen fibers were determined by modified Ashcroft scoring (28–30). Which is a numerical scale (from 0-5) for determining the fibrosis degree in lung specimens. Whereas positive immunoreactions of TNF-α and NF-κB were detected in the immunostained sections (31).

#### 2.5.3 Biochemical assays

Each left lung was homogenized with a homogenizer (IKA Ultra–Turrax T 25 Basic, Germany) using 4 volumes of ice-cold (50 mM, pH 7.4) Tris-HCl buffer containing Triton X-100 (0.50 mL/L) at 13,000 rpm for 2 min. The homogenates were centrifuged at 5000 x g for 20 min to eliminate debris. The supernatants were separated for further biochemical analysis (1). The activities of the oxidative stress markers malondialdehyde (MDA), superoxide dismutase (SOD), and reduced glutathione (GSH) were determined in the lung homogenates to assess the oxidative stress condition (1, 32). Inflammatory marker levels, including (IL-1β, GM-CSF, and TNF-α) in lung tissue, were measured using enzyme-linked immunosorbent assay-based kits (My BioSource, CA, USA) at absorbance 450 nm. In each sample, the amounts of IL-1β, GM-CSF, and TNF-α were quantified against their respective standard curves. On the basis of the conversion of biliverdin to bilirubin, lung tissue hemeoxygenase-1 (HO-1) activity was determined. The quantity of bilirubin produced was calculated by subtracting the optical densities (OD) at 460 and 530 nm. One unit of HO-1 activity is equal to the quantity of bilirubin (in nanomoles) generated per hour per mg of protein.

#### 2.5.4 Assessment of the mRNA levels of inflammatory cytokines and antioxidants

The left lung pieces were stored at −80°C in RNA later™ stabilization solution (Ambion, Inc. Thermo Fisher Scientific). In lung tissue homogenates, the mRNA levels of the inflammatory cytokines granulocyte colony-stimulating factor (*GM‐CSF*), tumor necrosis‐alpha (*TNF‐α*), interleukin‐beta (*IL‐1β*), as well as the glutathione peroxidase 2 (*GPx-2*), antioxidants hemoxygenase‐1 (*HO‐1*) and Nuclear factor erythroid 2-related factor 2 (*Nrf2*) were assessed by reverse transcriptase-polymerase chain reaction (RT-PCR). RNeasy Mini Kit (Qiagen, CA, USA) was used to extract the total RNA from the lung homogenates. The complementary DNA (cDNA) was prepared by a QuantiTect Reverse Transcription Kit (Qiagen, CA. USA) from 1 μg of total RNA. RT-PCR was performed in a 96‐well plate format on a CFX96 RT-PCR system (Bio‐Rad, Hercules, CA, USA). The PCR-reaction mixture included cDNA (equivalent to 100 ng of total RNA), master mix (SYBR Green-Kappa Bioscience, MA, USA), and 10 pmol each of the forward and reverse primers for *TNF-α*, *HO-1*, *GM‐CSF*, *Nrf2*, *GPx-2*, and *IL‐1β* (**Table S1** in the **Supplementary Material**). The thermal cycler adjusted as following: initial denaturation at 94°C for 5 min, 35 cycles of denaturation at 94°C for 15 Sec, annealing for 30 sec at 57°C, and elongation for 1 min at 65°C. The *GAPDH* gene was amplified alongside the target genes as an internal control. Each specimen was amplified three times. The data were analyzed using the 2-ΔΔCt method.

#### 2.5.5 Statistical analysis

The data are presented as the mean ± standard division, mean of eight rats/each group (n=8) and these means between the groups were assessed using the ANOVA test followed by the Tukey–HSD test. Data is tested for normality using Shapiro-Wilk test and it showed that data was normally distributed. Values of *p* ≤ 0.05 were considered to be indicative of statistical significance.

## 3 Results

### 3.1 HPLC analysis

**Figure 1:**
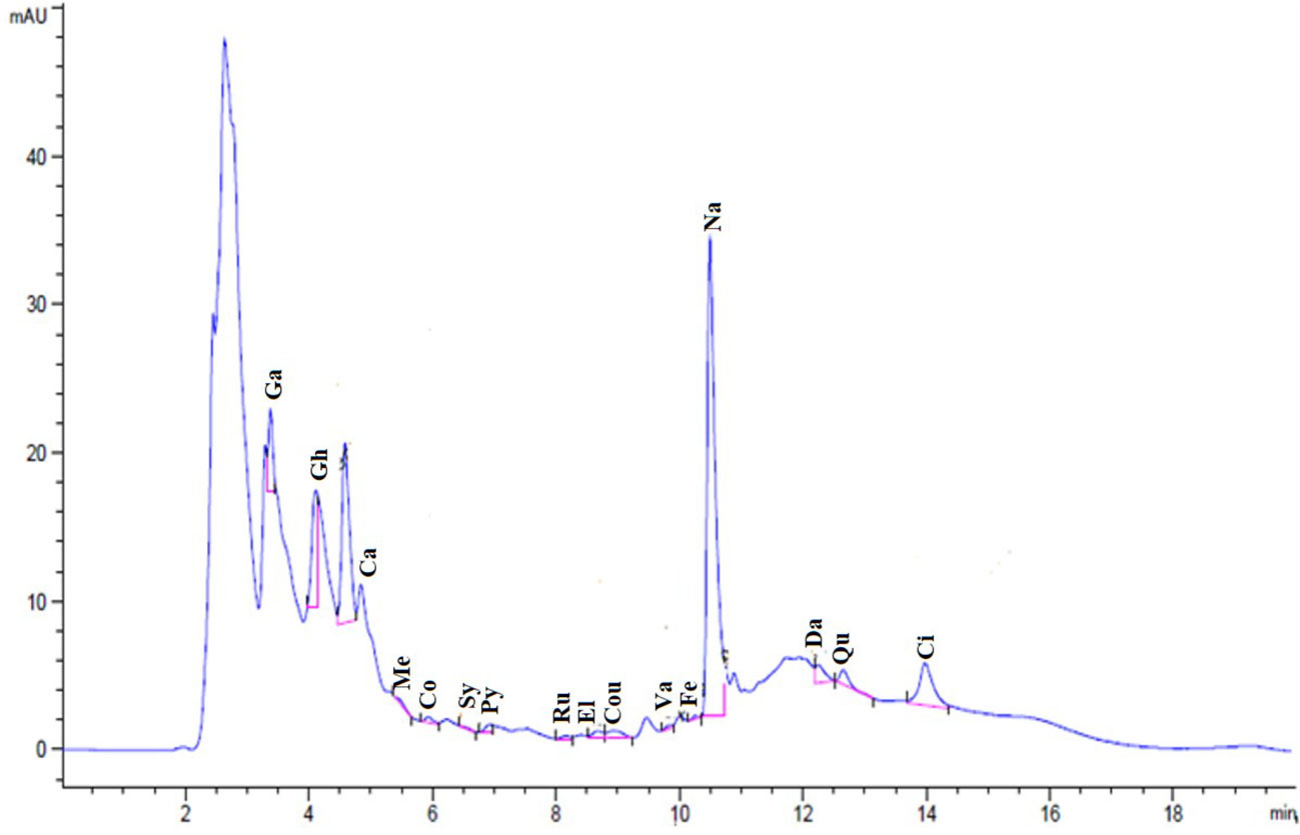
Chromatogram for the identified flavonoids and phenolic compounds of SE.

The chemical composition identification and quantification of methanol SE were performed using HPLC. **Figure 1** shows the HPLC chromatogram for the identified phenolic compounds and flavonoids in the SE. HPLC identified 16 compounds (**Table 1**). The most abundant phenolic compound was chlorogenic acid (7.34 μg/mL), and the major flavonoid was naringenin (30.19 μg/mL).

**Table 1:** HPLC analysis of the chemical composition of SE, Retention time (RT).

RT (Min)	Area (mAU*s)	Conc. (µg/ml)	Identified compound
3.38	25.80	2.07	Gallic acid
4.11	52.09	7.34	Chlorogenic acid
4.59	102.95	26.79	Catechin
5.5	2.19	0.14	Methyl gallate
5.93	3.34	0.29	Coffeic acid
6.57	1.09	0.11	Syringic acid
6.94	4.24	0.56	Pyro catechol
8.16	1.71	0.23	Rutin
8.68	5.82	1.02	Ellagic acid
8.94	8.24	0.24	Coumaric acid
9.86	1.93	0.11	Vanillin
10.25	1.38	0.11	Ferulic acid
10.5	297.11	30.19	Naringenin
12.25	13.07	0.89	Daidzein
12.65	9.67	1.19	Quercetin
13.98	45.03	1.08	Cinnamic acid

### 3.2 Histopathological findings

**Figure 2:**
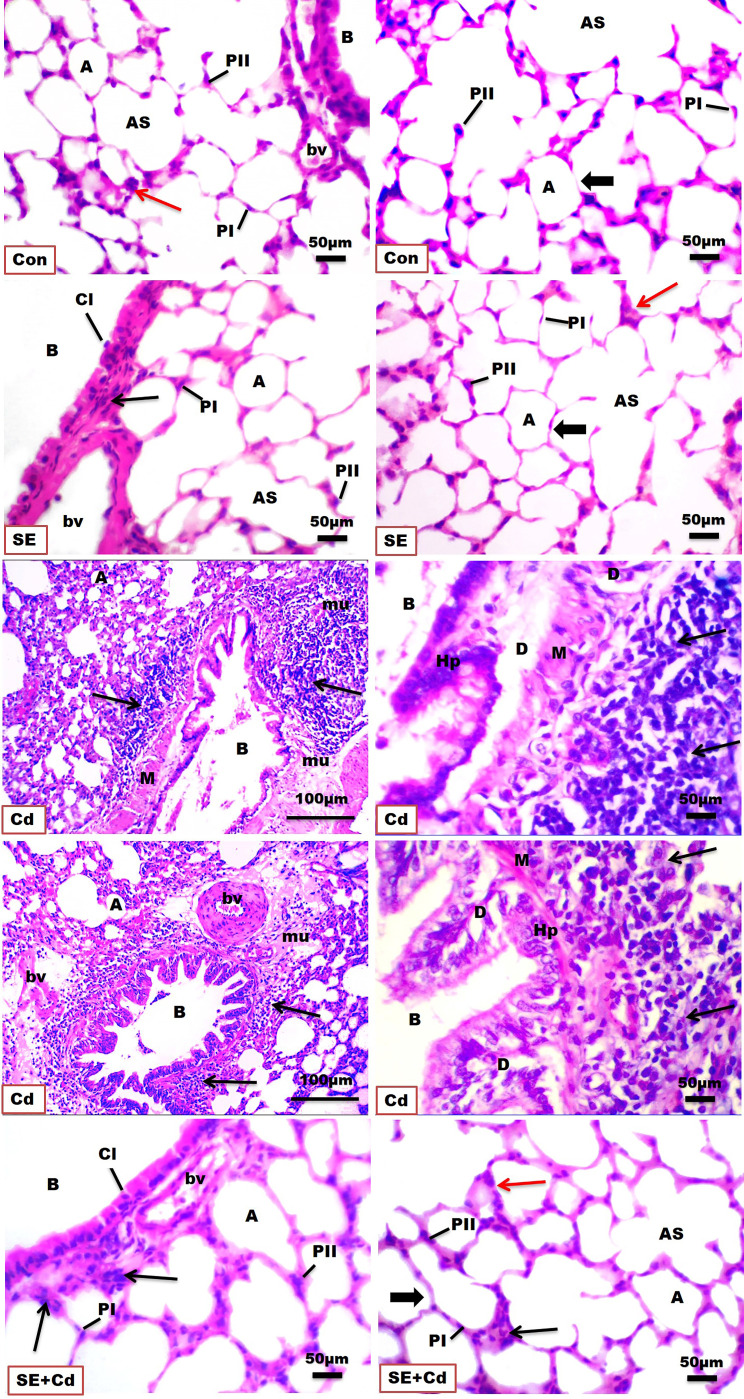
Photomicrographs of H&E-stained lung sections of the controls (Con), strawberry extract (SE), cadmium (Cd), and strawberry extract+cadmium (SE+Cd) rats show the histological architecture and histopathological alterations including alveolus (A) separated by interalveolar septa (thick arrow), type I pneumocytes (PI), type II pneumocytes (PII), alveolar sac (AS), bronchiole (B), inflammatory cell (Cl), cytoplasmic vacuolization (CV), hyperplastic degenerated bronchial epithelium (D), smooth muscle (M), alveolar macrophage (red arrows) inflammatory cells populations adjacent to bronchioles and alveolar ducts (thin black arrow), edema with mucous and collagen (mu), blood vessel (bv), and capillary (C), (Bar = 100μm= X 10, and 50μm= X 40).

H&E-stained lung slices from both the control and SE groups showed normal histological architecture of the pulmonary parenchyma, including normal airways and blood vessels, and what seemed to be normal alveolar septa at the end of the research (**Figure 2**, **Table 2**). The bronchioles are lined by pseudostratified columnar epithelium with a few Clara cells, gradually becoming columnar and cubical in the small respiratory bronchioles. Smooth-muscle bundles, adventitia, and lamina propria surround the mucosa. The alveoli and alveolar sacs are bordered by two types of pneumocytes, type I with flattened nuclei and type II with rounded nuclei, as well as a few alveolar macrophages. In contrast, the Cd group showed massive degenerative inflammatory disorders in the bronchiolar and alveolar epithelium with vacuolated cytoplasm, hemorrhage, marked obstruction of alveoli, and disrupted and thickened interalveolar septal cells in addition to enlarged blood vessels. This thickening was apparent because of significant inflammatory edema, mucus, and collagen fibers combined with congestion in the interalveolar septa and extensive inflammatory cell infiltration proximal to the bronchioles and alveolar ducts. Other sections showed hyperplastic degenerated bronchial epithelium combined with an increased number of alveolar macrophages (dust cells) and inflammatory cell aggregations with alveolar epithelial hyperplasia-filled and -replaced alveoli. In contrast, the lining epithelial layers of the bronchi in the SE+Cd group were almost identical to those of controls, with few inflammatory cells seen around the bronchi. Remodeling of the alveolar septa was also observed and was characterized by a clean and empty lumen, a single row of pneumocytes, and a reduction in the number of inflammatory cells.

**Table 2:** The histological and immunohistochemical evaluation of lung sections from the different groups under study.

Groups Scores	Con	SE	Cd	SE+Cd
**Total lung injury**	0	+1	+4	+1
**Airway inflammation**	+1	+1	+5	+2
**Alveolar edema**	0	+1	+4	+1
**Fibrosis**	+1	+1	+4	+2
**Positive IHC of TNF-α**	+1	+1	+4	+2
**Positive IHC of NF-κB**	+1	+1	+4	+2

Control (Con), strawberry extract (SE), Cadmium (Cd), strawberry extract+cadmium (SE+Cd), tumour necrosis‐alpha (TNF‐α), and nuclear factor kappa-light-chain-enhancer of activated B cells (NF-κB), extensive or severe alterations (+5), increased diffuse alterations (+4), moderate changes (+3), mild alterations (+2), minimal changes (+1) and no alterations (0), n= 8 for each group.

### 3.3 Masson’s trichrome staining

**Figure 3:**
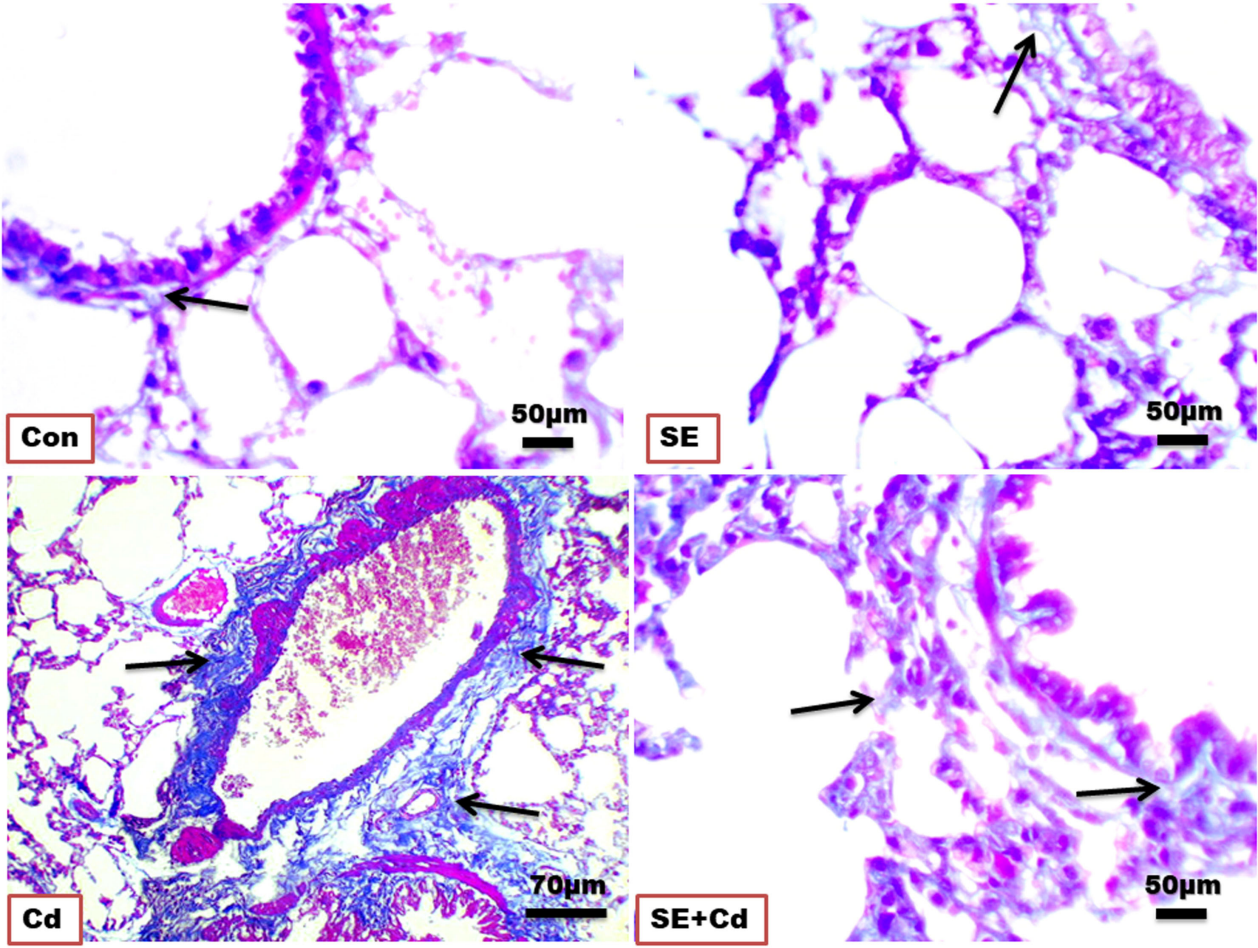
Photomicrographs of lung sections of the control (Con), strawberry extract (SE), cadmium (Cd), and strawberry extract+cadmium (SE+Cd) rats show a slight, minimal, strong, and mild blue-green collagen fibers expressions (arrows), respectively, Masson trichrome stain (Bar = 50μm= X 40).

Lung sections (**Figure 3**) showed blue staining indicating normal interstitial collagen fibers (1.583 ± 0.037%) in the interalveolar septa, lamina propria, and adventitia surrounding the bronchioles in the control group, and little expression (1.995 ± 0.087%) in the SE group in the lamina propria and around the wall of bronchioles. The lung sections of the Cd-exposed group showed an increase in blue collagen deposition (17.223 ± 3.918%) in the interalveolar septa and around the bronchioles and blood vessels, indicating interstitial fibrosis. Simultaneously, sections of the SE+Cd group showed slightly-evident interstitial collagen (4.897 ± 1.487%) in the interalveolar septa as well as around the bronchi wall (**Figure 3**). The lung collagen appearing as blue- to green-stained areas was analyzed according to modified Ashcroft score (**Table 2**).

### 3.4 Toluidine blue staining

**Figure 4:**
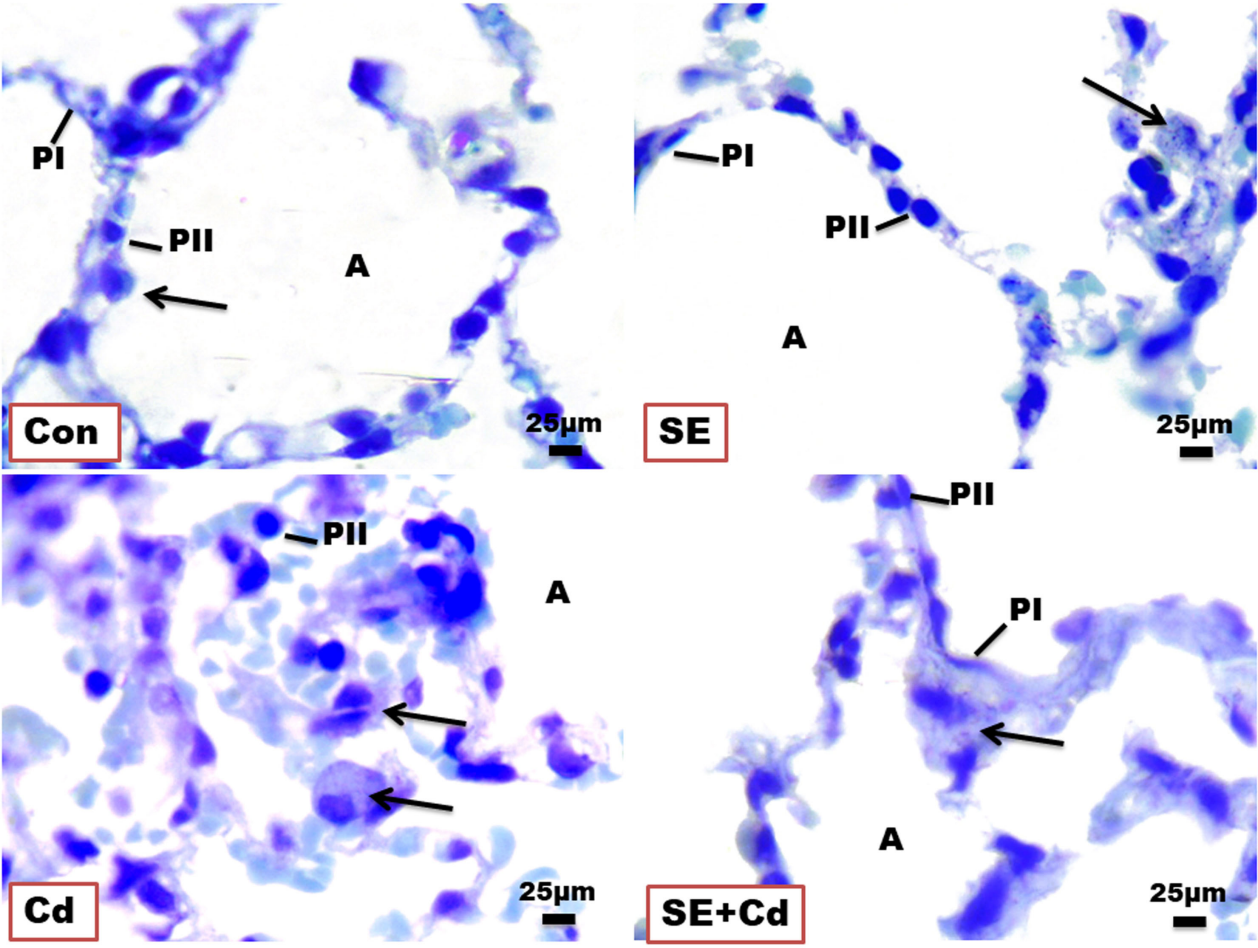
Photomicrographs of lung semithin sections of the control (Con), strawberry extract (SE), cadmium (Cd), and strawberry extract+cadmium (SE+Cd) rats show alveolus (A), type I pneumocyte (PI), type II pneumocyte (PII), and alveolar macrophage (arrows), Toluidine blue stain (Bar = 25μm= X 100).

Lung slices from the Control and SE groups revealed two epithelial cell types in the normal alveolar wall (Pneumocystes Type I and II) in the presence of blood capillaries for gas exchange. On the other hand, the Cd group showed alveolar epithelium degeneration with an increased number of macrophages. Rats from the SE+Cd group had a nearly restored normal alveolar epithelium structure (**Figure 4**).

### 3.5 Ultrastructural photomicrographs

**Figure 5:**
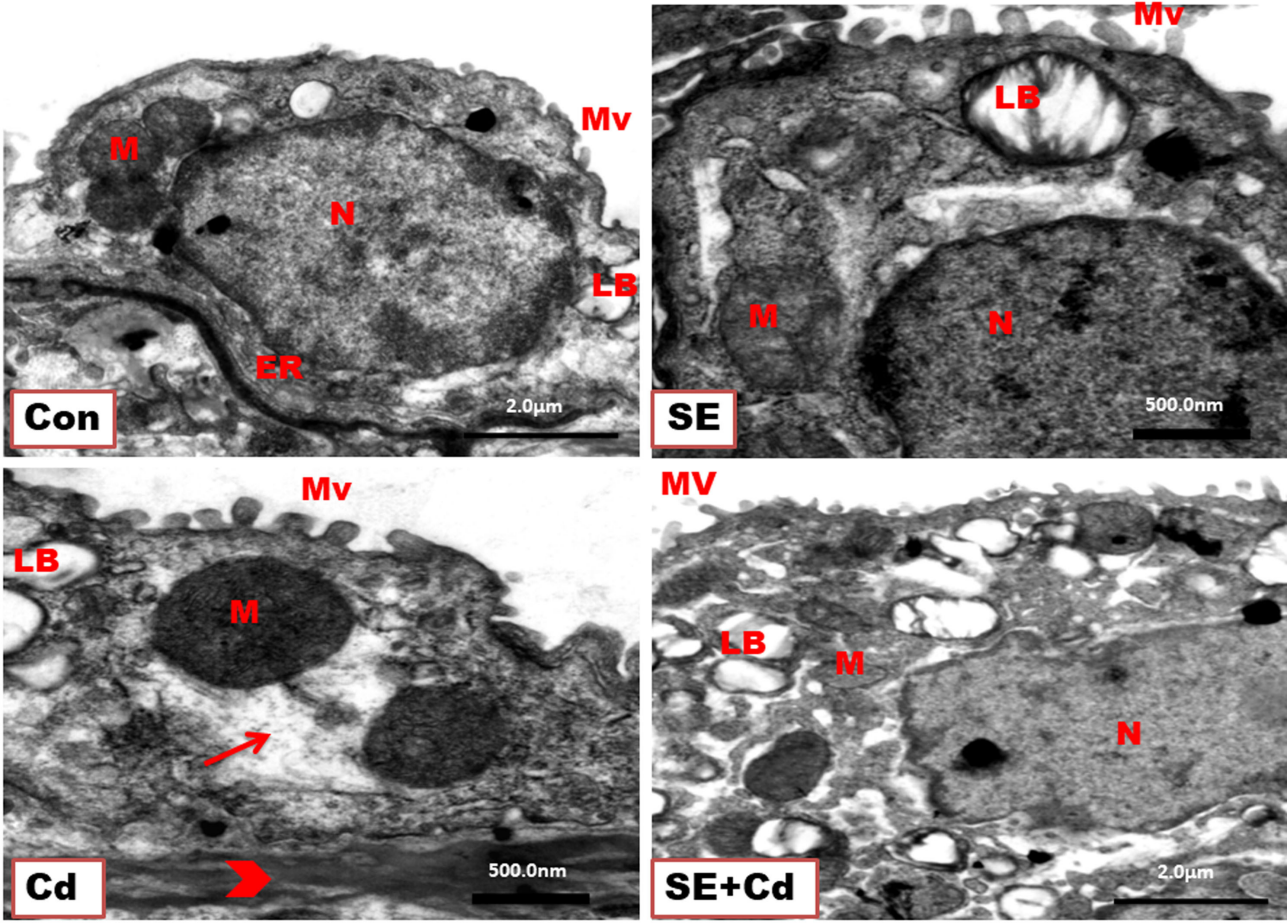
TEM of type II pneumocytes from lung ultrathin sections of the control (Con), strawberry extract (SE), cadmium (Cd), and strawberry extract+ cadmium (SE+Cd) rats show nucleus (N), mitochondria (M), endoplasmic reticulum (ER), lamellar bodies (LB), short microvilli (Mv) on the free surface, degeneration (arrow), and thick alveolar septum (head arrow), uranyl acetate–lead citrate double stain (Bar = 500nm, and 2μm).

**Figure 5** shows the normal type II pneumocytes found between type I pneumocytes in the alveoli of lung ultrathin sections from different groups. They appeared as large-cuboidal cells with a few short apical microvilli, lamellar bodies containing a phospholipid surfactant in their cytoplasm, central nuclei, abundant rough endoplasmic reticulum, well-developed Golgi apparatus, and mitochondria in lung ultrathin sections from the Control and SE group rats. In contrast, type II pneumocytes from the Cd group rats showed damaged lamellar bodies, enlarged swelled mitochondria, degenerated cytoplasm, and thick alveolar septum. Lung sections from the SE+Cd group revealed that the most changes observed in the Cd group had dissipated but haven’t completely disappeared.

### 3.6 SE immuno-attenuated Cd‐induced lung inflammation

**Figure 6:**
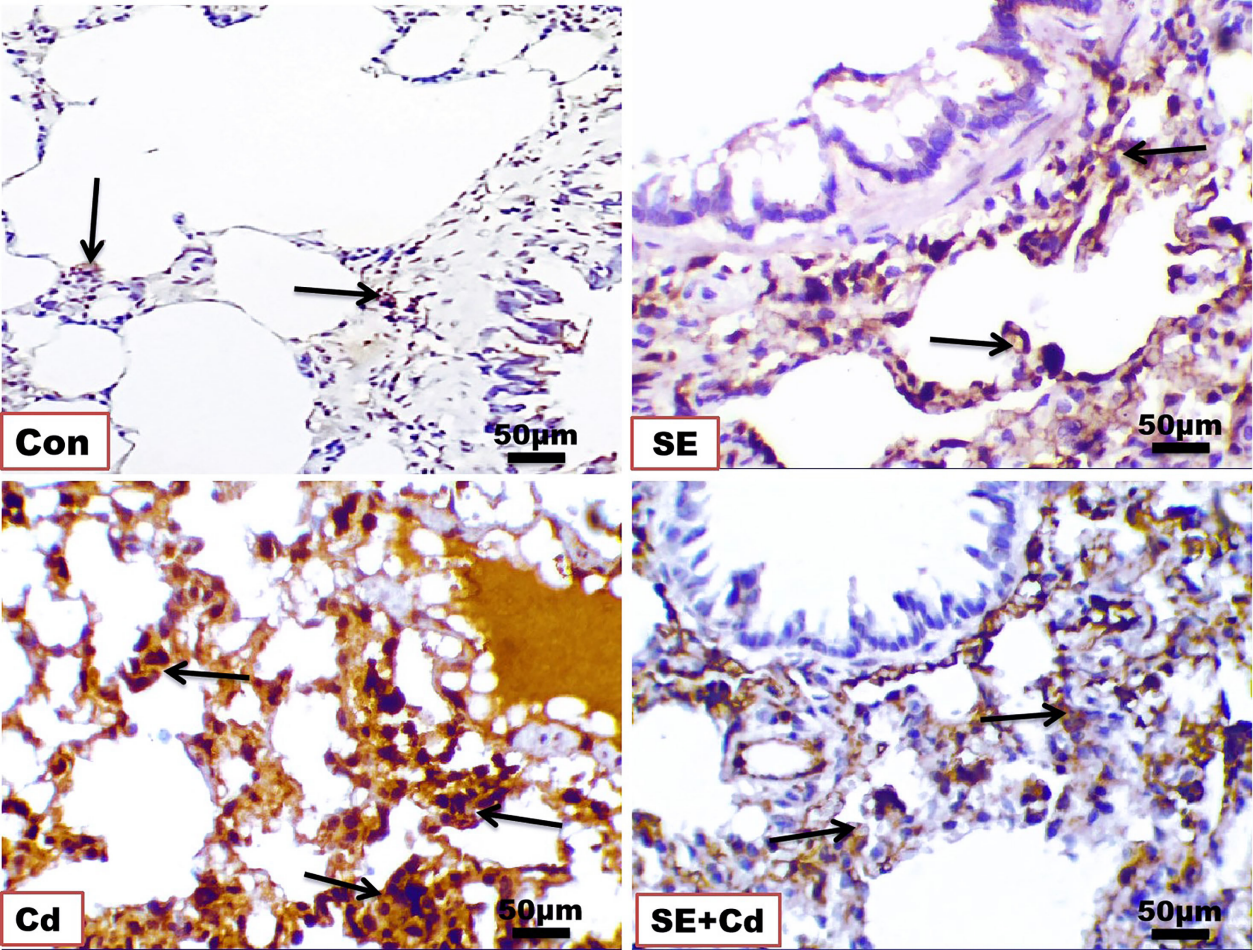
Photomicrographs of lung immuno-sections of the control (Con), strawberry extract (SE), cadmium (Cd), and strawberry extract+cadmium (SE+Cd) rats show brown tumor necrosis‐alpha (TNF-α) immuno-expression (arrows), immunohistochemical stain (Bar = 50μm= X 40).

**Figure 7:**
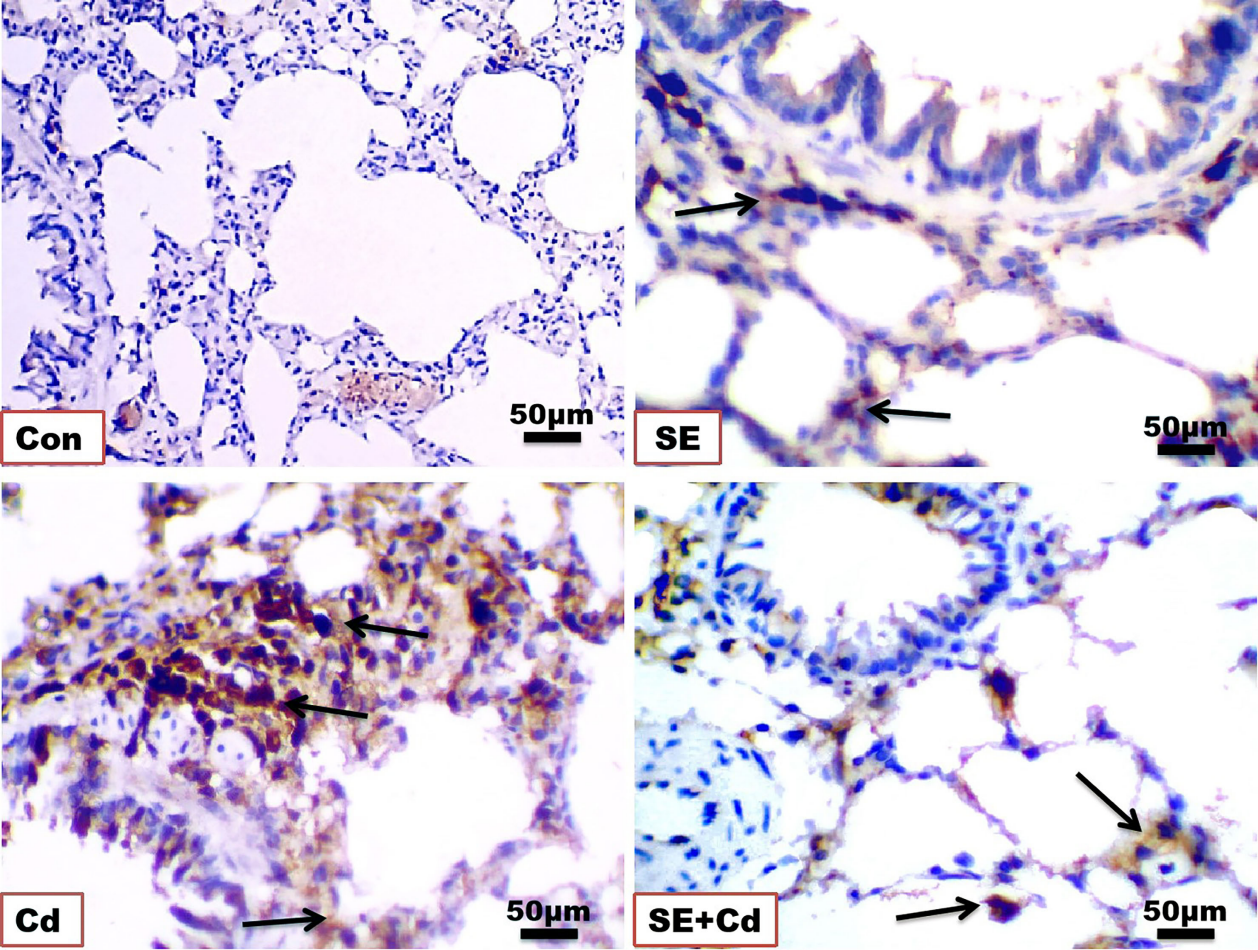
Photomicrographs of lung immuno-sections of the control (Con), strawberry extract (SE), cadmium (Cd), and strawberry extract+cadmium (SE+Cd) rats show NF-κB expression (arrows), immunohistochemical stain (Bar = 50 μm = X 40).

The immunohistochemical analysis of TNF-α and NF-κB in lung sections (**Figures 6**, **7**) from the control group showed a slightly positive (0.308 ± 0.075%, 0.912 ± 0.325%, respectively) brown immune reaction in the alveoli. However, some cells with positive immune reactions were seen in interalveolar septa in the SE group (2.144 ± 0.466%, 0.968 ± 0.468%, respectively). The majority of cells with strong positive TNF-α and NF-κB (21.065 ± 3.716%, 24.364 ± 2.129%, respectively) immune reactions were seen inside the alveoli and blood vessels in the septa in the Cd group. Sections of the SE+Cd group showed few cells with mid-positive immune reactions for TNF-α and NF-κB (4.077 ± 0.896%, 3.532 ± 0.307%, respectively). These observations are supported by the histologic results in **Table 2**.

### 3.7 SE suppressed oxidative stress in Cd‐treated rats

For SE antioxidant evaluation, SOD, MDA, and GSH were determined in the lung homogenates of the different groups. The results showed non-significant differences between controls and SE-supplemented rats in lung MDA, SOD, and GSH. On the other hand, the levels of MDA showed a significant rise in the Cd-treated rats and a significant reduction in both SOD and GSH activities relative to those in the controls (*p* < 0.05). SE+Cd-treated rats exhibited a statistically significant decrease in MDA levels and a significant increase (*p* < 0.05) in both SOD and GSH activities. TNF‐α, GM‐CSF, IL‐1β, and HO‐1 were significantly decreased in the Cd group. All of these parameters were nearly restored to semi-normal values after SE+Cd treatment in contrast to the values in the rats exposed to Cd alone (**Table 3**).

**Table 3:** Lung oxidative stress markers, inflammatory markers and antioxidant enzyme activities in all groups.

Groups Parameters	Con	SE	Cd	SE+Cd
**SOD (U/mg)**	11.2 ± 3.11**^#^**	11.9 ± 2.1 **^ns #^**	5± 0.96*^#^	7.8 ± 1.8*
**GSH (nmol/mg)**	21.7 ± 3.61	22.02 ± 3.7**^ns^**	12.5 ± 3.19*^#^	17.3 ± 1.92 ^ns^
**HO-1** **(nmol bilirubin/h/mg)**	4.9 ± 1.32	4.7 ± 1.29 **^ns^**	3.5 ± 1.22*^#^	5.2 ± 1.8 ^ns^
**MDA (nmol/mg)**	1.2 ± 0.56**^#^**	1.4 ± 0.47 **^ns #^**	5.2 ± 1.48*^#^	3.7 ± 0.51*
**GM‐CSF (pg/mg)**	2.09 ± 0.69**^#^**	2.18 ± 0.62 **^ns #^**	6.7 ± 1.27*^#^	4.17 ± 0.91*
**TNF‐α (pg/mg)**	142± 12.7**^#^**	147 ± 46.4 **^ns #^**	452 ± 11.5*^#^	327 ± 63.5*
**IL‐1β (ng/mg)**	42.1 ± 6.6**^#^**	40.8 ± 5.91 **^ns #^**	81± 11.28*^#^	58 ± 4.6*

Values are presented as the mean ± standard division, mean of eight rats/each group (n=8) and these means between the groups were assessed using the ANOVA test followed by the Tukey–HSD test. Control (Con), Strawberry extract (SE), Cadmium (Cd), Strawberry extract+Cadmium (SE+Cd), superoxide dismutase (SOD), reduced glutathione (GSH), hemoxygenase‐1 (HO‐1), malondialdehyde (MDA), granulocyte colony-stimulating factor (GM‐CSF), tumour necrosis‐alpha (TNF‐α), interleukin‐beta (IL‐1β). Note: (**^ns^**) indicates insignificance compared to the control, (*) indicates a significance compared to the control and SE groups, while (**^#^**) indicates a significance compared to the SE+Cd group, p < 0.05.

### 3.8 Inflammatory and antioxidant mediator mRNA levels

**Figure 8:**
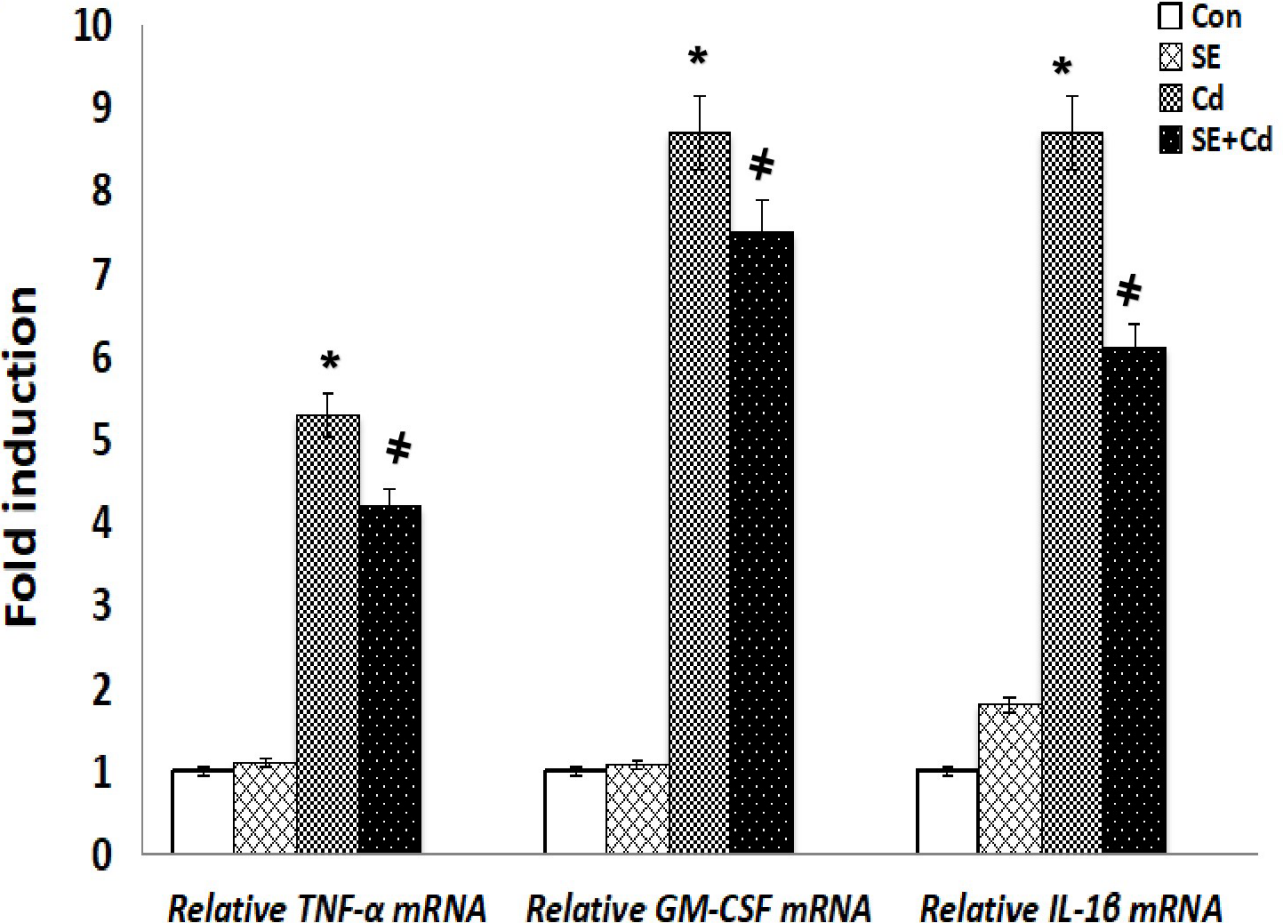
Schemes of *TNF-α*, *GMF-αe* and *ILnd e*mRNA relative levels in lung tissues of control (Con), strawberry extract (SE), cadmium (Cd)‐exposed, and SE+Cd‐treated rats. Values are presented as the mean ± standard division, mean of eight rats/each group (n=8) and these means between the groups were assessed using the ANOVA test followed by the Tukey–HSD test, (*) indicates a significance compared control vs. Cd, while ^(#)^ indicates a significance compared Cd vs SE+Cd, *p* < 0.05.

**Figure 9:**
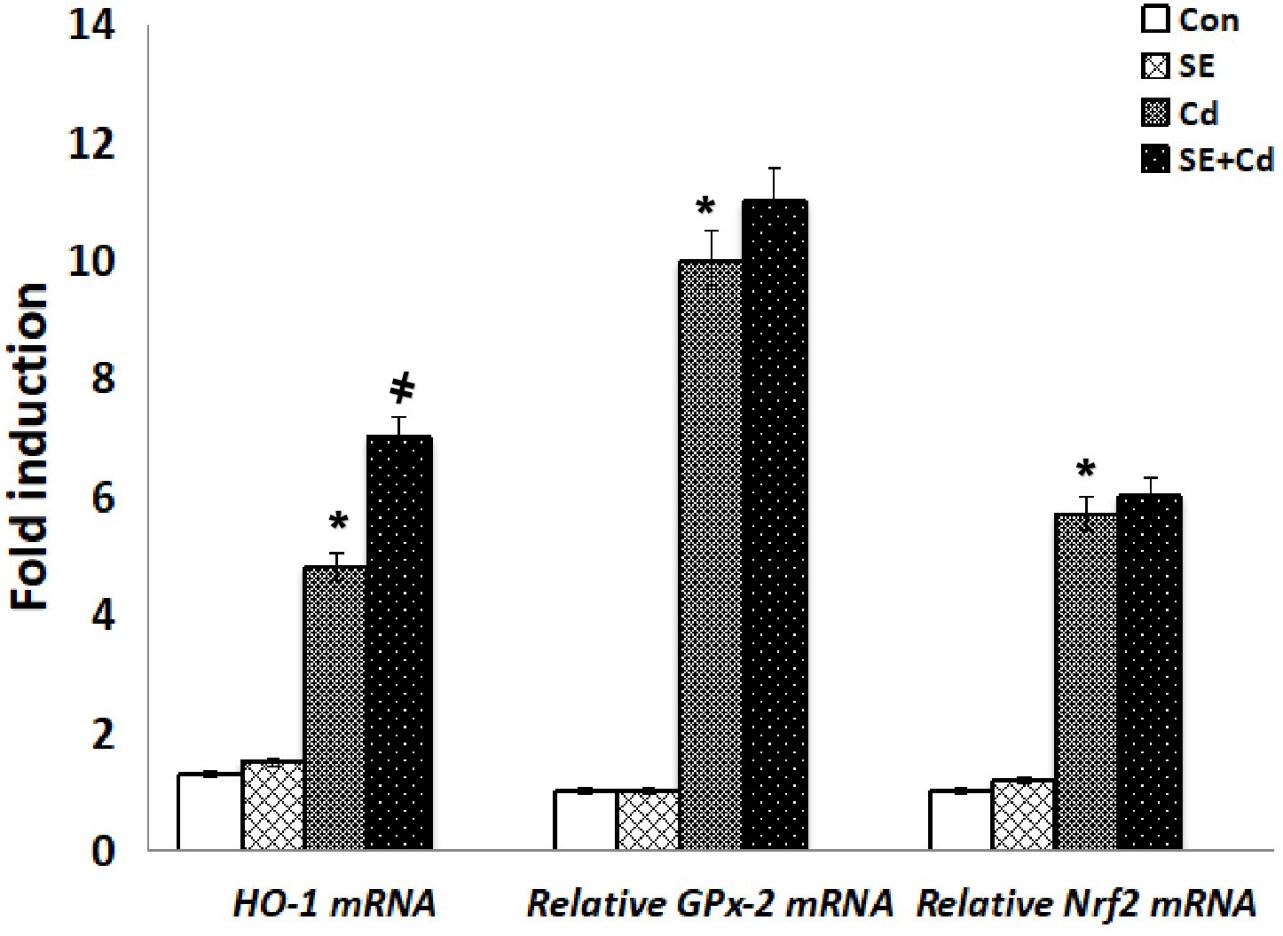
Schemes of *HO-1*, *GPx-2*, and *Nrf2* mRNA relative levels in lung tissues of controls, strawberry extract (SE), cadmium (Cd)-exposed and SE+Cd-treated rats. Values are presented as the mean ± standard division, mean of eight rats/each group (n=8) and these means between the groups were assessed using the ANOVA test followed by the Tukey–HSD test, (*) indicates a significance compared control vs. Cd, while ^(#)^ indicates a significance compared Cd vs SE+Cd, *p* < 0.05.

RT-PCR was performed to assess *GM‐CSF*, *IL‐1β*, and *TNF-α* expression at the mRNA level (**Figure 8**). The inflammatory cytokine mRNA levels were significantly upregulated in the Cd‐exposed rats compared with those in the SE‐treated rats or controls. *TNF-α*, *GM-CSF*, and *IL-1β* mRNA levels had decreased considerably in the SE+Cd group compared with the Cd group. RT-PCR was performed to investigate *GPx-2*, *HO-1*, and *Nrf2* expression at the mRNA level (**Figure 9**). The *HO-1*, *GPx-2*, and *Nrf2* mRNA levels were significantly upregulated in the Cd group relative to the control rats. Antioxidant parameter mRNA levels increased dramatically in the SE+Cd group and varied significantly between the SE-treated and control rats.

## 4 Discussion

Cadmium bio-accumulation in the living system can harm the reproductive system, gastrointestinal tract, mucous tissues, and neurological system severely. Inhalation is a common route of cadmium (Cd) exposure, and the lung is one of the primary target organs of Cd toxicity. Pulmonary inflammation, typified by elevated levels of chemokines and pro-inflammatory cytokines, appears to have a role in the development of cd-induced lung damage (33). Herbal plants are becoming increasingly popular as health promoters, while fruits and vegetables have long been used as medicines since they are inexpensive and have little adverse effects on animals and people. Strawberry (*Fragaria ananassa*) is one of the world’s most popular fruits. Diverse clinical manifestations are induced in human due to lung injuries. Therefore, the use of medicinal plant extracts is urgently needed to treat the disease because of their bioactive compounds. Thus the present research was done to estimate the ameliorative effects of the strawberry extract against pneumonia in an animal model. The study revealed several histopathological, ultrastructural, immunohistochemical, biochemical, and molecular disorders in rat lungs after Cd administration. The cadmium dose in the research (2 mg/kg of rat body weight) is regarded an average that did not cause any mortality in Cd-inoculated rats and is based on earlier studies that evaluated the toxic impact of cadmium in rats at doses ranging from 1 to 5 mg/kg (34–38). The SE treatment 1 hour before Cd exposure reduced Cd toxicity by significantly ameliorating the inflammation and oxidative stress. Pretreatment 1 hour of *Fragaria ananassa* extract is also based on prior research that looked at the preventive effect of *Fragaria ananassa* distinct herbal extract against cadmium poisoning (34, 36, 39–41). The protective effect of *Fragaria ananassa* methanolic extract was previously tested in a rat model of cadmium chloride-induced hepatotoxicity and neurotoxicity (2, 40) and proved that the *Fragaria ananassa* methanolic extract protected liver and brain tissue from Cd-induced hepatic and neuronal toxicity by improving the antioxidant system and increasing antiapoptotic and anti-inflammatory activities, and this previous result supports the current findings. Notably, SE reversed the increase in inflammatory cell populations and cytoplasmic vacuolization of the epithelium, disrupted thickened interalveolar septa, enlarged blood vessels, increased collagen fibers, led to positive immuno-expressions for *TNF-α* and *NF-κB*, elevated *MDA*, *GM-CSF*, *TNF-α*, and *IL-1β* levels, and reduced *GSH*, *SOD*, *HO‐1*, and *Nrf2* levels in Cd‐exposed rats. In the Cd-exposed group, interstitial fibrosis, chronic inflammation, injury to type I pneumocytes, and hyperplasia of type II pneumocytes with loss of their microvilli were observed. These results were consistent with many previous studies (2, 41–43). Bronchiolar and alveolar epithelium hyperplasia and cytoplasmic vacuolization occurred concurrently with or secondary to inflammation, and infiltrating cells, such as neutrophils, alveolar macrophages, and lymphocytes, were observed in the Cd-treated lungs, which was evidence of edema, suppurative inflammation, or hyperemia (4, 44). Cytoplasmic vacuolization is one of the degenerative changes associated with tissue damage. It represents the distended mitochondrial, pinched-off endoplasmic reticulum, and damaged lysosomes, which led to insufficient beta-oxidation and decreased levels of protein synthesis, lipid transport, and lipid accumulation within the organelles or cytoplasm. These signs had already been confirmed by the ultrastructure results in this research and agreed with (45). Previously (4), and (41) revealed Cd-induced redox cycling cytotoxicity in pneumocytes type II. In the current study, severe interstitial, blue-stained collagen replaced the Cd-treated lung parenchyma, indicating lung fibrosis. Lung fibrosis often occurs as focal fibrous tissues in the airway lumens, a common response to lung damage, and is frequently associated with inflammation (44, 46). reported that oral fisetin administration as a strawberry flavonol revealed resistance to chronic airway disease in mice. Polyphenol agents, such as those in date palm, have been shown to significantly ameliorate cytotoxicity by improving antioxidant and immunity alterations in rat testes (47, 48).

Lung tissue is one of the primary targets of Cd poisoning (49), and inhaling Cd-contaminated air has severe effects on the respiratory system. As a result of Cd-induced pneumonitis, shortness of breath, edema of the lungs, and mucosal damage have been noted (50). Humans and experimental animals exposed to Cd developed lung damage, pulmonary fibrosis, emphysema, and inflammation (44). It has been shown that Cd affects the lungs by reducing cell viability and impairing lung cell function (51). Inflammation and oxidative stress generated by Cd are responsible for lung injury, producing proinflammatory cytokines by lung epithelial cells and destroying the extracellular matrix (52).

Cells exposed to sustained oxidative stress and inflammation may generate tissue damage (53, 54). Lymphocyte infiltration, edema, lymphocyte aggregation, thickening of interalveolar septa, enlargement of specific air spaces, dilatation, and congestion of the pulmonary vein were indicative of severe inflammation in the lung tissue (53). It has been suggested that Cd-mediated oxidative stress induced these changes (4, 55).

Mulberry and strawberry fruit polysaccharides reduce alveolar cell injury (56). The present data revealed that the preventive effect of strawberries on Cd-induced lung toxicity involved alleviating lung damage. Because strawberries are abundant in phenols, which include hydroxyl groups that form coordinated bonds with Cd2+, SE treatment avoided this lung damage by chelating Cd. These findings support the hypothesis that SE protects the lung from oxidative damage caused by CdCl_2_ and can prevent lung pathogenesis (57). The current ameliorative effect of SE may be attributable to its content of several antioxidant components, such as phenolic acids, and flavanols (Catechins), which identified as strong antioxidant constituents of berry fruits (15, 16, 58). Berry fruits consuming, such as strawberries, has been shown to protect from oxidative stress-related disorders, such as cancer and diabetes (2, 59, 60). Some phenolic compounds in strawberries are believed to have contributed to their biological effects by scavenging free radicals (61). However, it is unknown if strawberries can mitigate the harm caused by heavy metals. This study examined the possible impact of SE on the pulmonary damage caused by Cd in rats. These studies indicated that the detrimental effects of Cd might be attributable to its ability to create ROS, which trigger cell damage, including DNA mutations, protein degradation, and death (62). In addition, Cd interacts with metal cofactors in various enzymatic and non-enzymatic antioxidants to decrease SOD and GSH levels (63). It has been shown that Cd harms numerous organs via oxidative stress, inflammation, leukocyte infiltration, and proinflammatory effects (64–67).

In the current study, treatment with SE was associated with a significant reduction in *TNF-α*, *GM-CSF*, and *IL-1β* mRNA levels relative to those in Cd-exposed rats. The authors check the inflammasome’s activation through measuring the activity of pro-inflammatory cytokines interleukin-1β (IL-1β). Metabolic abnormalities caused the production of inflammasomes. Following the formation of these protein complexes, the inflammasomes activate caspase 1, which proteolytically activates the pro-inflammatory cytokines interleukin-1 (IL-1 β) and IL-18 (68). This effect as attributed to the SE being rich in phenolic compounds, including some flavonoids (69, 70), which has been associated with immuno-modulation of the pro-and anti-inflammatory cytokine secretions (71, 72). In the lung tissue of rats, the levels of *TNF-α*, *GM‐CSF*, and *IL‐1β* rose significantly following intravenous injection of 1 mg/kg Cd. *TNF* has been hypothesized to be a key mediator in the development of Cl2-caused toxicity (73). In addition, these results were compatible with those of many study findings that SE inhibited the translocation of *NF-κB* and *GM-CSF*, decreasing release of proinflammatory cytokines, such as *IL-1β* and *TNF-α* (74). Moreover, SE downregulated the expression of *NF-κB* and *GM-CSF*, leading to downregulation of proinflammatory cytokines, such as *IL-1β* and *TNF-α*, demonstrating the anti-inflammatory activity of strawberries (70, 75, 76). Another explanation for the anti-inflammatory effect of SE is its immunomodulatory effect on T-helper type 1/T-helper type 2, which are responsible for the expressions of some cytokines, such as *TNF-α*, *GM-CSF*, and *IL-1β* (56). This study found that SE significantly increased the mRNA levels of antioxidant parameters, such as *HO-1*, *GPx-2*, and *Nrf2*, indicating its potent antioxidant properties (70, 77). The antioxidant effect of SE could be explained by its capacity to reduce oxidative stress and scavenge different ROS, such as superoxide radicals, hydrogen peroxide, hydroxyl radicals, and singlet oxygen (2, 61). Another study found that SE increased the serum antioxidant capacity significantly in humans, which was detected by different approaches, including the assay of oxygen radical absorbance capacity, Trolox-equivalent antioxidant capacity (TEAC), and ferric-reducing ability (78). Another study revealed that SE had the highest TEAC because of its high anthocyanin content (79).

Anthocyanins, which are one of the main bioactive component in dietary berries, especially strawberries, justify investigating their effects on these antioxidant biomarkers, such as the activities of SOD and GPx-2 enzymes that were significantly increased after consumption of strawberries (80), as noticed in the present research. Anthocyanins from strawberries exhibit antioxidant activity via various biochemical processes, including the activities of anthocyanin-induced antioxidant enzymes that generate antioxidants via the *Nrf2* pathway and reduce inflammation. Additionally, anthocyanins inhibit glutathione depletion and maintain GST activity normal. The levels of *Nrf2*, *NADPH*, quinine oxidoreductase-1, and *HO-1* were also raised. It can be concluded that SE induced antioxidant defense through the *Nrf2* pathway and decreased inflammation by *NF-κB* inhibition (81). *Nrf2* inhibits the *NF-κB* pathway via increasing *HO-1* and antioxidants, which efficiently neutralizes oxidative stress and detoxifies chemicals thus decreases ROS mediated *NF-κB* activation (82).

## 5 Conclusions

This study showed that SE delivered 1 hour before Cd exposure protected the lungs of rats from CdCl_2_-induced pulmonary damage. The antioxidant and anti-inflammatory effects of the extract, which are probably mediated via the presence of polyphenols and flavonoid constituents, may be responsible for these positive effects. Therefore, SE may ameliorate against Cd-induced pulmonary damage. The authors recommend further large-scale clinical studies in human consuming strawberry rich diet to affirm these promising results in pneumonia and to test the application of strawberry use in other types of inflammatory lung diseases such as bronchitis and chronic obstructive pulmonary diseases.

### 5.1 Limitations and advantages

The advantages of this study, to the best of our knowledge, it was the first study that test the lung protective effect of *Fragaria ananassa* extract against cadmium induced lung toxicity. Despite the current study’s limitations (insufficient time of extract administration and the unavailability of using different doses of *Fragaria ananassa* extract), we recommend large scale randomized studies including bigger size of sample and more extended administration period to affirm these promising results which can be applied to different lung diseases. In addition, we recommend in the future to study the effect of *Fragaria ananassa* extract in human patient with different lung diseases such as pneumonia and bronchitis.

## Data availability statement

The datasets presented in this study can be found in online repositories. The names of the repository/repositories and accession number(s) can be found in the article/**Supplementary Material**.

## Ethics statement

The animal study was approved by the animal care and use committee (IACUC, Approval No. MUFS /F/HI/6/20), Menoufia University, Egypt, which followed the National Institutes of Health Guide for Care and Use of 424 laboratory animals (NIH publication No. 8023, received 1978). The study was conducted in accordance with the local legislation and institutional requirements.

## Author contributions

AN: Conceptualization, Data curation, Formal Analysis, Funding acquisition, Methodology, Project administration, Resources, Software, Supervision, Visualization, Writing – original draft. HA: Data curation, Funding acquisition, Methodology, Validation, Visualization, Writing – review & editing. RF: Funding acquisition, Validation, Visualization, Writing – review & editing. RE: Funding acquisition, Validation, Writing – review & editing. NO: Funding acquisition, Investigation, Visualization, Writing – review & editing. SE: Funding acquisition, Investigation, Writing – review & editing. AE: Data curation, Formal Analysis, Funding acquisition, Methodology, Resources, Software, Visualization, Writing – original draft.
