# Supplementary material for: Immunostimulatory and anti-inflammatory impact of Fragaria ananassa methanol extract in a rat model of cadmium chloride-induced pulmonary toxicity
Source: Front Immunol. 2023 Nov 29;14:1297315. doi: 10.3389/fimmu.2023.1297315 (PMC10716534; doi:10.3389/fimmu.2023.1297315)
Supplement: Supplementary file 1 [file DataSheet_1.pdf]

Supplementary Material

Table S1: Sequences of Primers of Genes applied in the current study.

Gene	Primer sequence	References
**GAPDH**	Forward :5′- CCTTCATTGACCTCAACTAC-3′<br>Reverse:5′-TTCACACCCATCACAAAC-3′	(Okamoto et al. 2001)
**TNF-alpha**	Forward:5′-CGGAAAGCATGATCCGAGAT-3′<br>Reverse:5-′: AGACAGAAGAGCGTGGTGGC-3′	(Li et al. 2020)
**GM-CSF**	Forward:5′-AGACCCGCCTGAAGCTATACAA-3′<br>Reverse:5′-CTGGTAGTGGCTGGCTATCATG-3′	(Żelechowska et al. 2021)
**GPx2**	Forward:5′-ACCGATCCCAAGCTCATCAT-3′<br>Reverse:5′-TCTCAAAGTTCCAGGACACATCTG-3′	(Bardag-Gorce et al. 2011)
**IL-1β**	Forward:5′- CCAGGATGAG GACCCAAGCA -3′<br>Reverse:5′-TCCCGACCAT TGCTGTTTCC -3′	(Siegling et al. 1994)
**HO-1**	Forward: 5′-TTAAGCTGGTGATGGCCTCC -3′<br>Reverse: 5′-GTGGGGCATAGACTGGGTTC-3′	(Fathi et al. 2020)
**Nrf2**	Forward:5′-GCCAGCTGAACTCCTTAGACTCA-3′<br>Reverse:5′-GCTCTGCTAGGAAAGCAGAGTAAAATT-3′	(Bardag-Gorce et al. 2011)

Bardag-Gorce, F.; Oliva, J.; Lin, A.; Li, J.; French, B. A.; French, S. W. Proteasome inhibitor up regulates liver antioxidative enzymes in rat model of alcoholic liver disease. Exp Mol Pathol. 2011, 90(1):123-130. DOI: 10.1016/j.yexmp.2010.10.013

Fathi, R.; Nasiri, K.; Akbari, A.; Ahmadi-KaniGolzar, F.; Farajtabar, Z. Exercise protects against ethanol-induced damage in rat heart and liver through the inhibition of apoptosis and activation of Nrf2/Keap-1/HO-1 pathway. Life Sci. 2020, 256:117958. DOI: 10.1016/j.lfs.2020.117958

Li, B.; Cheng, Z.; Sun, X.; Si, X.; Gong, E.; Wang, Y.; Tian, J.; Shu, C.; Ma, F.; Li, D.; Meng, X. Lonicera caerulea L. Polyphenols Alleviate Oxidative Stress-Induced Intestinal Environment Imbalance and Lipopolysaccharide-Induced Liver Injury in HFD-Fed Rats by Regulating the Nrf2/HO-1/NQO1 and MAPK Pathways. Mol Nutr Food Res. 2020, 64(10):e1901315. DOI: 10.1002/mnfr.201901315

Okamoto, K.; Martin, D.P.; Schmelzer, J.D.; Mitsui, Y.; Low, P.A. Pro- and anti-inflammatory cytokine gene expression in rat sciatic nerve chronic constriction injury model of neuropathic pain. Exp Neurol. 2001, 169(2):386-391. DOI: 10.1006/exnr.2001.7677

Siegling, A.; Lehmann, M.; Platzer, C.; Emmrich, F.; Volk, H.D. A novel multispecific competitor fragment for quantitative PCR analysis of cytokine gene expression in rats. J Immunol Methods. 1994, 177(1-2):23-28. DOI: 10.1016/0022-1759(94)90139-2

Żelechowska, P.; Brzezińska-Błaszczyk, E.; Różalska, S.; Agier, J.; Kozłowska, E. Mannan activates tissue native and IgE-sensitized mast cells to proinflammatory response and chemotaxis in TLR4-dependent manner. J Leukoc Biol. 2021, 109(5):931-942. DOI: 10.1002/JLB.4A0720-452R
